# The rising cases of diphtheria in Pakistan: A new epidemic boiling?

**DOI:** 10.7189/jogh.13.03027

**Published:** 2023-05-26

**Authors:** Waleed Inayat Mohamed, Muhammad Umar

**Affiliations:** 1 University of Health Sciences, Pakistan; 2 Khairpur Medical College, Pakistan

## CURRENT SITUATION

The number of sporadic cases of diphtheria in Pakistan is rising fast, as reported by an advisory issued by the country’s National Institute of Health (NIH), and health care professionals are alarmed [1]. By December 2022, 39 children and teenagers were reported dead, rising to over 45 by January 2023 [2,3]. Thirty-nine additional probable disease cases were reported just in one week of December 2022 [4]. The number of existing cases remain unclear due to the country’s inadequate health care infrastructure. The NIH recommends a three-dose diphtheria vaccine in a pentavalent form and a fourth booster dose for all children as part of the Expanded Program of Immunization in Pakistan [1]. The country’s vaccination rates have increased steadily over the decade, but the COVID-19 lockdown reversed this trend. In 2020, the United Nations Children's Fund (UNICEF) highlighted a seven per cent drop in childhood vaccination rates in Pakistan, increasing the number of unprotected children to 1.4 million [5]. Recent floods in the country also exacerbated the problem [3].

## WHY DOES PAKISTAN MATTER?

Pakistan ranks third globally in the number of unvaccinated children and is one of the only two countries in the world where polio still exists [4,6]. After investing billions of rupees in health care infrastructure, cases are at a record low, but sporadic ones still appear. Eight confirmed cases were reported in December from North Waziristan [4]. Pakistan is already suffering from endemic cases of extensively drug-resistant (XDR) typhoid, malaria, tuberculosis, cholera, and other disease which hinder vaccination efforts in the country. In 2021, 2274 cases of XDR typhoid were reported in the Southern province of Sindh [4]. Furthermore, illiteracy, fear of vaccines, parental disbelief, cultural myths, logistical hurdles, and incompetent attitudes have hindered mass vaccination [6], including for polio, diphtheria, and COVID-19. Diphtheria spikes have been reported in previous years, but none were related to the reduced immunisation rates as this one. With the increased incidence of diphtheria and its novel resistance to antibiotics, a potential epidemic is imminent [3], unless action is taken immediately.

## SITUATION DURING COVID-19 LOCKDOWN

The COVID-19 lockdown led to a drastic decrease in immunisation levels. In Karachi, the country’s most populous city, there was a 52.8% decrease in daily immunisation visits, with approximately 2734 children being left out of the EPI program daily [7]. A study from the northern province of Khyber Pakhtunkhwa found an average 36% decrease in immunisation rates during the COVID-19 lockdown [8]. Similarly, 18% of parents delayed their children’s vaccinations in Azad Kashmir [9]. Diphtheria is a vaccine-preventable disease, which implies that reduced vaccination coverage will lead to an increased incidence, which is true for Pakistan. Though treatable with antitoxins and antibiotics, supply disruption and poor-quality treatment due to the COVID-19 lockdown may have caused the recent outbreak.

## HOPE IS STILL THERE

In mid-2022, data released by Global Alliance for Vaccines and Immunisation (Gavi), showed a substantial increase in post-pandemic vaccination coverage. Diptheria tetanus pertussis (DTP3) coverage in Pakistan increased from 77% in 2020 to 83% at the end of 2021, reducing the number of zero-vaccinated children to 610 564. This was comparable to the pre-pandemic figures of 84% coverage in 2019 [4]. COVID-19 lockdowns and social distancing, while decreasing immunisation rates, led to a reduced spread of the much-feared diseases. Health leaders and their international partners played an effective and necessary role in achieving these results [4]. Nonetheless, waves of COVID-19 hits have disrupted health systems and recovery requires significant efforts.

**Figure:**
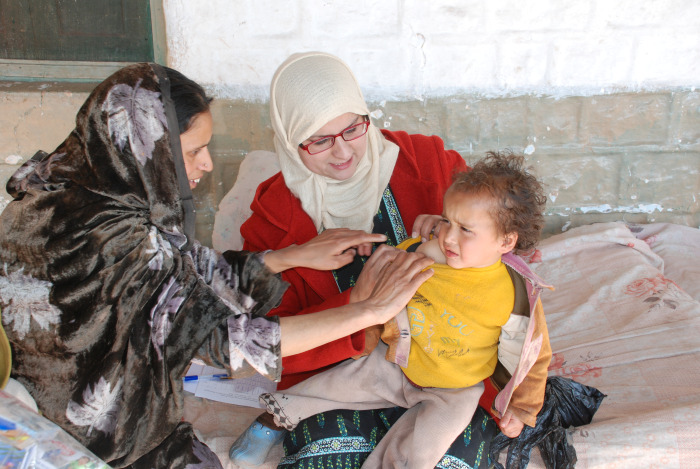
Photo: Pakistan vaccination campaign by CDC Global. Source: Available under CC BY 2.0 at: https://flic.kr/p/XqjpFw.

Highlighting this issue with the international audience, including medical practitioners and policymakers, is crucial in acting at both national and international levels. We still have time to respond. This phenomenon was observed not only in Pakistan, but globally, especially in third-world countries, where immunisation levels decreased sharply due to COVID-19, with most countries still being unable to bridge these gaps [4].

Pakistan’s current financial, economic, and political situation puts it in the spotlight. A country on the brink of debt default due to non-existent dollar reserves, affected by climate change and water scarcity, and prone to natural calamities, has relied on international support for immunisation and disease eradication. If a diphtheria epidemic strikes, it could spread into the population, burdening the already defunct healthcare system, affecting hundreds of millions of people and killing thousands. Non-availability of health services in the country’s peripheral regions, including areas in Khyber Pakhtunkhwa and interior Sindh, makes them most prone to these epidemics; these regions have been affected by pandemics more than any other region in Pakistan in history.

The unrestricted cross-border movement between Pakistan and Afghanistan has led to the perseverance of polio and may still provide a pathway for the diphtheria epidemic to spread and infect millions in the region.

Consequently, this pandemic should serve as a reminder to national immunisation programs to address disruptions in routine immunisation – otherwise secondary outbreaks of vaccine-preventable diseases may occur. Awareness and education about the benefits of immunisation are key to improvement.
